# Cerebral Autosomal Dominant Arteriopathy with Subcortical Infarcts and Leukoencephalopathy (CADASIL) with Aortic Dissection

**DOI:** 10.7759/cureus.72852

**Published:** 2024-11-01

**Authors:** Koji Hayashi, Hina Hamada, Mamiko Sato, Asuka Suzuki, Yuka Nakaya, Kazuyoshi Hosomichi, Yasutaka Kobayashi

**Affiliations:** 1 Department of Rehabilitation Medicine, Fukui General Hospital, Fukui, JPN; 2 Department of Rehabilitaion Medicine, Fukui General Hospital, Fukui, JPN; 3 Health Science, Fukui Health Science University, Fukui, JPN; 4 Laboratory of Computational Genomics, School of Life Science, Tokyo University of Pharmacy and Life Sciences, Tokyo, JPN

**Keywords:** acute cerebral infarction, aortic dissection (ad), cerebral autosomal dominant arteriopathy with subcortical infarcts and leukoencephalopathy (cadasil), large vessel, notch3 gene, small vessel, vascular dissection

## Abstract

This report describes the case of a 48-year-old Japanese man with cerebral autosomal dominant arteriopathy with subcortical infarcts and leukoencephalopathy (CADASIL) who also presented with aortic dissection. The patient had a history of hypertension, diabetes mellitus, dyslipidemia, and mild renal failure but had not received any treatment. He developed back pain and was diagnosed with type A aortic dissection via contrast-enhanced chest computed tomography (CT). He was treated with total arch aortic graft replacement. On the seventh day post-surgery, a brain CT revealed a cerebral infarction in the left frontal cortex, which was considered a surgical complication. T2-weighted fluid-attenuated inversion recovery (FLAIR) brain magnetic resonance imaging (MRI) performed five months after onset revealed widespread confluent white matter hyperintensities, including involvement of the bilateral temporal tips. Genetic testing identified a C457T mutation (p.R153C) in exon 4 of the NOTCH3 gene. Based on these findings, the patient was diagnosed with CADASIL. CADASIL is a monogenic inherited cerebrovascular small vessel disease and the leading genetic cause of subcortical stroke in adults. However, large vessel involvement related to CADASIL is less recognized. In this report, we discuss the relationship between CADASIL and aortic dissection.

## Introduction

Cerebral autosomal dominant arteriopathy with subcortical infarcts and leukoencephalopathy (CADASIL) is a monogenic inherited cerebrovascular small vessel disease (cSVD) and the leading genetic cause of subcortical stroke in adults [1]. The prevalence of CADASIL is estimated to be 1.3-5 per 100,000 [2]. CADASIL is pathologically characterized by diffuse angiopathy that is neither atherosclerotic nor amyloid, predominantly affecting small to medium-sized penetrating arteries and leptomeningeal arteries [3]. On the other hand, large vessel lesions related to CADASIL are less recognized. In this report, we describe a case with CADASIL accompanied by aortic arch dissection.

## Case presentation

**Figure 1:**
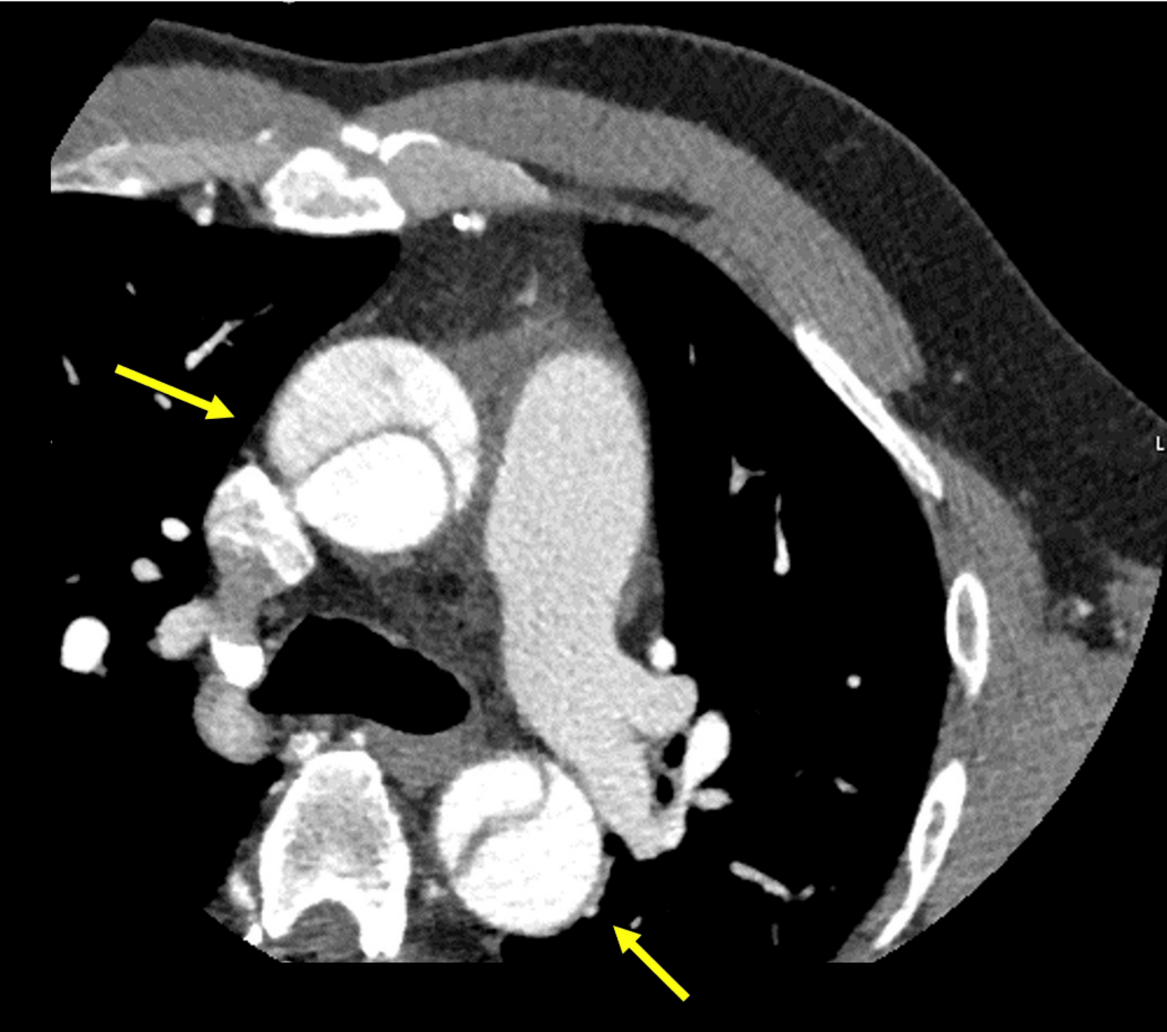
Chest contrast computed tomography (CT) scan results on admission The chest contrast CT shows arterial dissection extending from the ascending aorta to the aortic arch (arrowheads).

**Figure 2:**
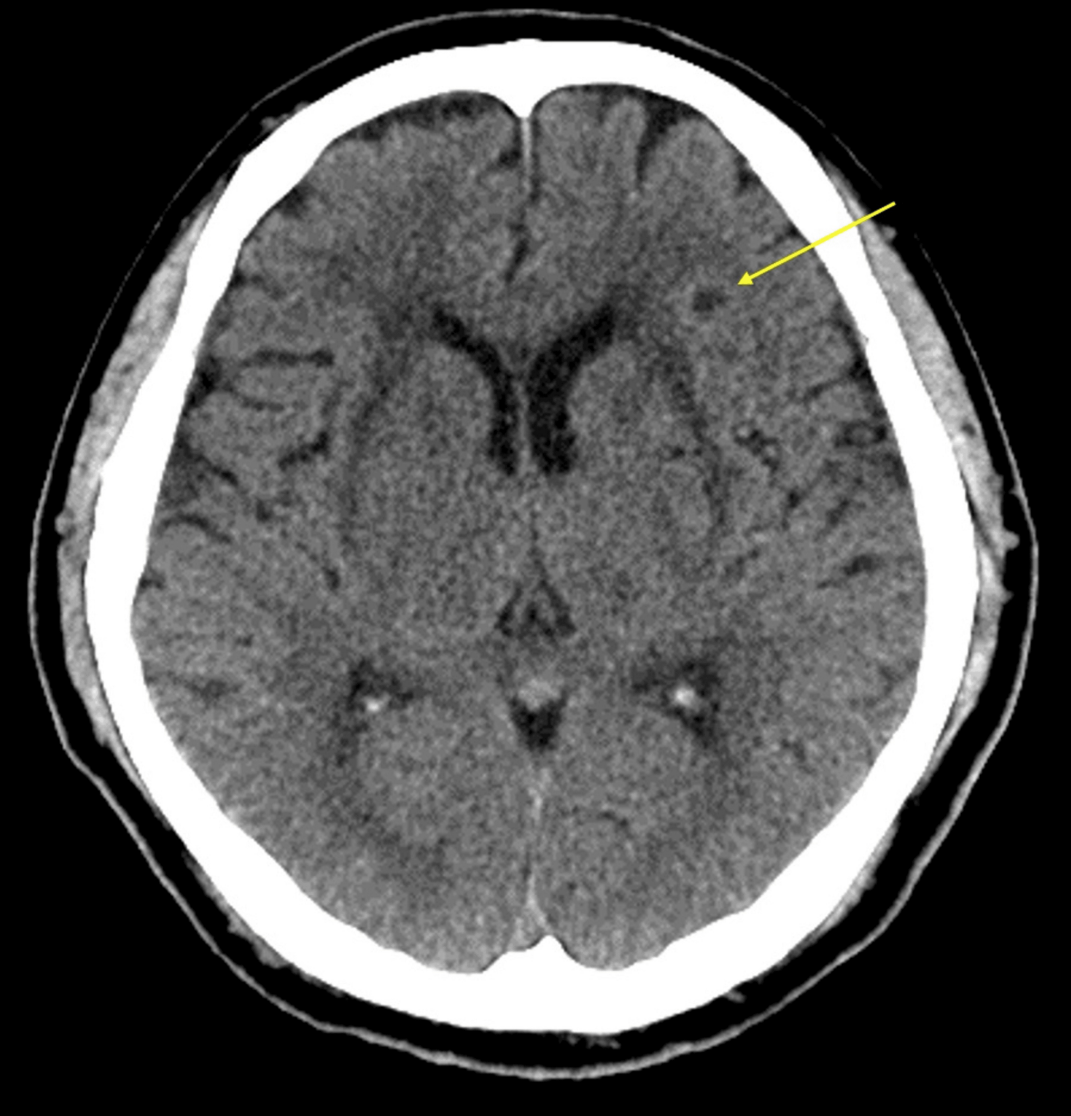
Brain CT results seven days after onset The brain CT shows scattered low-density areas in the left hemisphere (arrowhead).

A 48-year-old Japanese man developed back pain and perspiration and was transferred to the emergency room in a previous hospital admission. The patient had hypertension, diabetes mellitus, dyslipidemia, and mild renal failure during an annual medical check-up but had not received any treatment. He had no history of smoking and was an occasional drinker. Before this episode, he worked as a tax accountant and was independent in his activities of daily living (ADLs). His vital signs showed clear consciousness, a body temperature of 36.1°C, blood pressure of 156/90 mmHg, heart rate of 84/min, saturation of percutaneous oxygen (SpO₂) of 99%, and a respiratory rate of 22/min. Blood tests showed elevated white blood cells, aspartate aminotransferase, alanine aminotransferase, lactate dehydrogenase, creatine kinase, cholinesterase, fibrin degradation products, and D-dimer (Table 1). Chest contrast computed tomography (CT) scan revealed aortic dissection from the ascending aorta to the aortic arch (Figure 1). He was diagnosed with type A aortic dissection and treated with total arch aortic graft replacement. After surgery, delirium and difficulty awakening continued. Brain CT on day seven after the onset disclosed multiple cerebral infarctions (Figure 2).

**Table 1:** The result of blood tests on admission

Inspection items	Result	Reference range
White blood cell count	15600 /μl	3300–8600
Red blood cell count	541×10⁴ /μl	386–492×10⁴
Hemoglobin	15.6 g/dl	11.6–33.4
Hematocrit	47.9%	35.1–44.4
Blood platelet	17.9×10⁴ /μl	15.8–34.8
Total protein	8.6 g/dl	6.6–8.1
Albumin	4.9 g/dl	4.1–5.1
Total bilirubin	0.95 mg/dl	0.20–1.20
Direct bilirubin	0.24 mg/dl	0.00–0.40
Aspartate aminotransferase	53 U/l	13–30
Alanine aminotransferase	83 U/l	7–30
Lactate dehydrogenase	295 U/l	124–222
Creatine kinase	345 U/l	41–153
Creatine Kinase MB	18 U/l	0–24
Cardiac muscle troponin T	0.003 ng/ml	0–125
N-terminal pro-brain natriuretic peptide	39 pg/ml	0.000–0.100
Cholinesterase	457 U/l	185–450
γ-glutamyltransferase	59 U/l	11–64
Amylase	55 U/l	30–120
Blood urea nitrogen	12.0 U/l	8.0–20.0
Creatinine	0.95 mg/dl	0.46–0.79
Urine acid	6.4 mg/dL	3.0–7.0
Sodium	141 mmol/l	138–145
Potassium	3.4 mmol/l	3.6–4.8
Chlorine	104 mmol/l	101–108
Calcium	9.2 mg/dL	8.7–10.2
Magnesium	2.1 mg/dL	1.8–2.4
Hemoglobin A1c	6.1%	<5.5%
C-reactive protein	0.09 mg/dl	0.00–0.14
prothrombin time activity	104%	80–120
Prothrombin time-international normalized ratio	0.97	0.90–1.15
Activated partial thromboplastin time	25.1 sec	25.4–38.4
Fibrin degradation products	11.5 μg/ml	0.0–4.9
D-dimer	5.0 μg/ml	0.0–0.9

**Figure 3:**
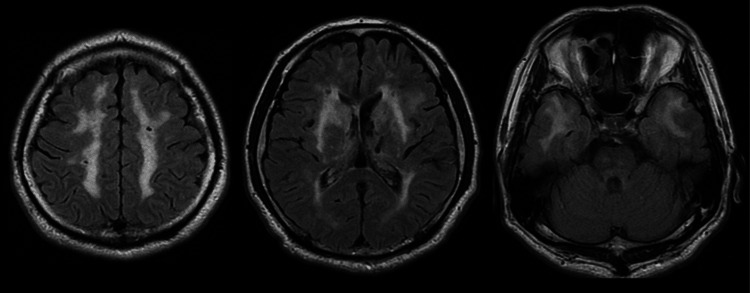
Brain magnetic resonance imaging (MRI) results five months after onset T2-weighted fluid-attenuated inversion recovery (FLAIR) brain MRI showing widespread confluent white matter hyperintensities, including involvement of the bilateral temporal tips.

Two months after the onset, he was transferred to our hospital for rehabilitation due to residual higher brain dysfunction. Neurological examinations on admission to our hospital revealed decreased activation, hoarseness, bilateral hypoacusis, and slight right hemiplegia (Brunnstrom stage: upper limb VI, hand VI, lower limb VI). Sensory disturbance, incoordination, and autonomic disturbance were not noted. Although he had a position of anteversion, his gait was normal. The Functional Independence Measure (FIM) score was 125. The patient's cognitive assessments yielded the following results: a score of 28 on the Hasegawa's Dementia Scale-Revised (HDS-R), 29 on the Mini-Mental State Examination (MMSE), and 17 on the Frontal Assessment Battery (FAB). T2-weighted fluid-attenuated inversion recovery (FLAIR) brain magnetic resonance imaging (MRI) performed five months after onset revealed widespread confluent white matter hyperintensities, including involvement of the bilateral temporal tips (Figure 3). Genetic testing identified a C457T mutation (p.R153C) in exon 4 of the NOTCH3 gene. Based on these findings, the patient was diagnosed with CADASIL. He continued rehabilitation therapy until 6.5 months after onset and was discharged from our hospital with near independence in ADLs. The patient then continued outpatient rehabilitation and was eventually able to return to his previous job.

## Discussion

This report describes a case of CADASIL with type A aortic dissection and cerebral infarction. T2-FLAIR brain MRI revealed widespread confluent white matter hyperintensities, and genetic testing identified a c.457C>T mutation (p.R153C) in exon 4 of the NOTCH3 gene, confirming the diagnosis of CADASIL. The p.R153C mutation is one of the major mutations associated with CADASIL [4]. In addition, c.457C>T mutation has been previously reported in China and Poland [5, 6]. Thus, this mutation is confirmed as pathogenic.

To the best of our knowledge, this is the first report about aortic dissection related to CADASIL. CADASIL is associated with the Notch3 protein, primarily expressed on vascular smooth muscle cells of blood vessels and pericytes [7]. This protein plays a crucial role in cellular communication [7]. Mutations in the NOTCH3 gene lead to the accumulation of misfolded Notch3 protein around small and medium-sized arteries throughout the body [7]. Consequently, CADASIL is generally thought to be linked to damage in small vessels, leading to cerebral small vessel disease (cSVD). Regarding the aorta involvements, Buczek et al. reported abdominal aorta aneurysm with CADASIL [8]. In this report, the authors described a familial occurrence of abdominal aortic aneurysms. In addition, Arboleda-Velasquez et al. reported that expression of the R1031C mutation in Notch3 triggered mural cell degeneration in the aortas in mouse models [9]. Thus, these findings suggest that CADASIL has some effect on the aorta and is related to the development of aortic aneurysms and other conditions. Arterial dissections in the middle cerebral artery and coronary artery have been reported in association with CADASIL. However, these previous reports do not address the etiology of the dissections in relation to CADASIL [10, 11]. It remains unclear whether CADASIL and aortic dissection are coincidental or related; based on previous reports, we believe that aortic dissection is not a chance occurrence but is associated with CADASIL.

His multiple cerebral infarctions were confirmed by CT seven days after total aortic arch replacement. Aortic dissection can lead to acute cerebral infarction, making thrombolytic therapy contraindicated in such cases [12]. A key characteristic of cerebral infarction associated with aortic dissection is left hemiplegia, typically resulting from reduced blood flow through the right carotid artery, which is the first branch of the aortic arch [13, 14]. As a result, cerebral infarctions in the regions supplied by the right carotid artery are common. In addition, a brain MRI study revealed that the actual incidence of cerebral infarction after thoracic endovascular aortic repair is 34.4% [15]. Moreover, CADASIL is also well-known to be associated with stroke [1]. In our case, the precise mechanisms behind the development of cerebral infarctions remain unclear; however, the postoperative cerebral infarction is most likely due to the timing of onset and the occurrence of the left-sided cerebral infarction.

## Conclusions

This report presents a case of CADASIL accompanied by type A aortic dissection. Based on data from previous literature, the combination of CADASIL and aortic dissection represents an association rather than a chance occurrence. Further studies are needed to uncover the underlying mechanisms of aortic dissection development in patients with CADASIL.
